# Genetic atypical hemolytic uremic syndrome in children: a 20-year experience from a tertiary center

**DOI:** 10.1590/2175-8239-JBN-2020-0199

**Published:** 2021-05-12

**Authors:** Cristiana Maximiano, Andreia Silva, Inês Duro, Tiago Branco, Liane Correia-Costa, Ana Teixeira, Liliana Rocha, Teresa Costa, Paula Matos, Maria do Sameiro Faria, Conceição Mota, Alberto Caldas Afonso

**Affiliations:** 1 Hospital de Braga, Departamento de Pediatria, Braga, Portugal.; 2 Centro Hospitalar Tondela-Viseu, Departamento de Nefrologia, Viseu, Portugal.; 3 Centro Hospitalar Universitário do Porto, Centro Materno-Infantil do Norte, Departamento de Pediatria, Porto, Portugal.; 4 Centro Hospitalar do Tâmega e Sousa, Departamento de Pediatria, Penafiel, Portugal.; 5 Centro Hospitalar Universitário do Porto, Centro Materno-Infantil do Norte, Unidade de Nefrologia Pediátrica, Porto, Portugal.; 6 Universidade do Porto, Instituto de Ciências Biomédicas Abel Salazar, Porto, Portugal.; 7 Universidade do Porto, Instituto de Saúde Pública, Porto, Portugal.; 8 Universidade Nova de Lisboa, REQUIMTE, Unidade de Investigação em Biociências Moleculares Aplicadas, Porto, Portugal.

**Keywords:** Atypical Hemolytic Uremic Syndrome, Child, Genetic Testing, Thrombotic Microangiopathies, Síndrome Hemolítico-Urêmica Atípica, Criança, Testes Genéticos, Microangiopatias Trombóticas

## Abstract

**Introduction:**

Atypical hemolytic uremic syndrome (aHUS) is a rare disorder characterized by the triad of microangiopathic hemolytic anemia, thrombocytopenia, and acute kidney injury, which primarily affects preschool-aged children. This study’s aim was to describe the clinical profile, management, and long-term outcome of the genetic aHUS patients admitted to a tertiary care pediatric nephrology center during 20 years.

**Methods:**

We performed a retrospective analysis of the clinical records of all aHUS patients younger than 18 years with identified genetic mutations. Data on clinical features, genetic study, therapeutic interventions, and long-term outcomes were reviewed.

**Results:**

Five cases of aHUS with an identified genetic mutation were included; all were inaugural cases with the youngest being 4 months old. Complement factor H gene mutation was identified in four patients. Therapeutic plasma exchange was performed for acute management in 4 patients, one of whom also needed acute renal replacement therapy (peritoneal dialysis). All patients went on complete remission, 2 had more than one relapse but only 1 of these progressed to chronic kidney disease during the follow-up period (median (25th-75th percentile), 136 (43.5-200.5) months).

**Conclusion:**

In children, the prognosis of renal function seems to be strongly dependent on the genetic background, thus being crucial to perform genetic study in all aHUS cases. In our cohort, 2 patients presented genetic mutations not previously described. Recent innovations on the genetic field leading to the identification of new mutations has lead to a better understanding of aHUS pathogenesis, but further studies, focusing on the genotype-phenotype correlation, with longer follow-up periods, are needed.

## Resumo

**Introdução:**

A síndrome hemolítica urêmica atípica (SHUa) é um distúrbio raro caracterizado pela tríade de anemia hemolítica microangiopática, trombocitopenia e lesão renal aguda, afetando principalmente crianças em idade pré-escolar. O objetivo deste estudo foi descrever perfil clínico, manejo e desfecho em longo prazo dos pacientes com SHUa genética admitidos em um centro terciário de nefrologia pediátrica durante 20 anos.

**Métodos:**

Realizamos análise retrospectiva dos registros clínicos de todos os pacientes com SHUa menores de 18 anos com mutações genéticas identificadas. Revisaram-se dados sobre características clínicas, estudo genético, intervenções terapêuticas e desfechos em longo prazo.

**Resultados:**

Incluíram-se cinco casos de SHUa com uma mutação genética identificada; sendo todos casos inaugurais, o mais jovem tendo 4 meses de idade. A mutação no gene do fator H do complemento foi identificada em quatro pacientes. Plasmaférese terapêutica foi realizada para tratamento agudo em 4 pacientes, um dos quais também necessitou terapia renal substitutiva aguda (diálise peritoneal). Todos os pacientes tiveram remissão completa, 2 mais de uma recidiva, mas apenas 1 evoluiu para doença renal crônica durante acompanhamento (mediana (percentil 25°-75°), 136 (43,5-200,5) meses).

**Conclusão:**

Em crianças, o prognóstico da função renal parece ser fortemente dependente do histórico genético, sendo crucial realizar estudo genético em todos os casos de SHUa. Em nossa coorte, 2 pacientes apresentaram mutações genéticas não descritas anteriormente. Inovações recentes no campo genético que levaram à identificação de novas mutações conduziram a um melhor entendimento da patogênese SHUa, mas são necessários mais estudos, focando na correlação genótipo-fenótipo, com períodos de acompanhamento mais longos.

## Introduction

Hemolytic uremic syndrome (HUS) is a rare disorder characterized by microangiopathic hemolytic anemia, thrombocytopenia, and acute kidney injury (AKI) secondary to thrombotic microangiopathy. Atypical HUS (aHUS) is distinguished from typical or Shigatoxin-producing *Escherichia coli O157:H7* (STEC) HUS by the absence of STEC infection. aHUS can be distinguished from thrombotic thrombocytopenic purpura (TTP) by a normal level of ADAMTS13 (a disintegrin and metalloprotease with thrombospondin type 1 motif, member13) activity^1^^,^ 2.

aHUS is a rare disorder with a reported annual incidence of 0.5 cases per million^3^, which can be idiopathic or secondary to potential triggers, such as upper respiratory tract infections, fever, pregnancy, drugs and non-STEC diarrheal illnesses. Extra-renal manifestations are reported to occur in around 20% of the cases, most commonly involving the central nervous system and presenting as altered state of consciousness, seizures or focal neurologic deficits, and the gastrointestinal tract, especially presenting with prodromic diarrhea (in around 30% of the patients). Nonspecific findings, such as hypertension and malaise, can also occur but are often related to the underlying renal involvement^3^^,^ 4. aHUS is associated with a dismal prognosis, a relapsing course, high acute mortality and frequent progression to end-stage renal disease (ESRD) 5.

In recent years, aHUS has been found to be associated with genetic or autoimmune abnormalities leading to dysregulation of the alternative complement pathway on the surface of the vascular endothelium^1^^,^ 3. In almost 60% of aHUS patients, mutations in genes encoding complement-regulating proteins are reported, either resulting in loss of function in a complement regulatory gene or in gain of function in an effector gene^1^^,^ 6. Mutations in 6 genes have been associated with increased susceptibility for aHUS - complement factor H (CFH), complement factor B, complement factor I, membrane cofactor protein (MCP), C3, and thrombomodulin. The screening for diacylglycerol kinase ( (DGK() mutation should also be performed in children especially in those with age of onset before 1-2 years. According to 2015 international consensus approach, genetic screening should be performed in all cases of aHUS (first episode or relapse), in case of familial history of non synchronous aHUS, pregnancy/post-partum aHUS, or in case of *de novo* post-transplant aHUS^7^.

Therapeutic plasma exchange (TPE) was the mainstay of treatment for aHUS until 2014, when new data reveled considerable morbidity associated with plasma therapy in children along with eculizumab approval. Since then, several studies have demonstrated that effective terminal complement blockade with eculizumab can rescue native kidney function and allow successful kidney transplantation after renal function loss due to aHUS^6^^,^ 7.

aHUS emerged throughout recent years as a new disorder and then a few studies have come along the way. In the present study, we aimed to review all pediatric cases of aHUS admitted in our tertiary care pediatric nephrology center with causative genetic mutation identification, over the last 20 years, in order to characterize their clinical profile, management, long-term outcome, with particular focus on the relapse episodes and progression to chronic kidney disease (CKD).

## Methods

We performed a retrospective analysis of all pediatric patients with aHUS, with a genetic mutation identified, diagnosed and managed between 1999 and 2020 in the Pediatric Nephorology Unit at Centro Materno-Infantil do Norte, Centro Hospitalar Universitário do Porto, Portugal.

The diagnosis of aHUS requires the existence of the following features: i) microangiopathic hemolytic anemia, characterized by the elevation of serum lactate dehydrogenase level, notable decrease of serum haptoglobin level and the presence of schistocytes on a peripheral blood smear; ii) thrombocytopenia, and iii) AKI^1^^,^ 2. In pediatric patients, AKI is defined as a rise in the serum creatinine levels of at least 1.5 times the upper limit of the age and sex-specific pediatric reference range^4^.

A Next-Generation Sequencing (NGS) panel of 11 genes for aHUS was performed in 4 cases and 1 case (diagnosed before NGS implementation in our center) was only analyzed for MCP and CFH associated genes.

Data on baseline clinical findings (presence of fever, oligo/anuria, gastrointestinal symptoms, upper respiratory tract infections and hypertension), biochemical parameters (hemoglobin, platelets, lactate dehydrogenase, urea, creatinine, serum complement components C3 and C4 levels), direct coombs test, and acute management performed (renal replacement therapy (RRT), TPE and/or eculizumab treatment) was collected at patient admission with first aHUS episode. Data on follow-up, regarding long-term outcome, was also collected, namely occurrence of complete remission, need for TPE, relapses, development of hypertension, and evolution to CKD. Renal function was estimated through glomerular filtration rate calculation (eGFR) using the creatinine-based “Bedside Schwartz” equation (2009)^8^. Systolic and diastolic BP were classified according to the American Academy of Pediatrics criteria and hypertension was considered as SBP and/or DBP equal or above to the 95^th^ percentile for sex, age, and height^9^.

Considering the number of patients included in the present analysis, formal statistical analysis was not performed and only a descriptive analysis is presented.

## Results

A total of 5 children were included, 3 were male. The median (25^th^-75^th^ percentile, P25-P75) age of patients at the first episode of aHUS was 19 (10.5-41) months. One patient presented before 1 year of age, with 4 months of age. Demographic and clinical data of the included patients is presented in Table 1. Only 1 of our patients had family history of aHUS - his mother had history of cerebrovascular disease; all other 4 patients were considered sporadic aHUS cases.

**Table 1:** Acute management and long-term outcome of all included aHUS patients

	Patient 1	Patient 2	Patient 3	Patient 4	Patient 5
**Gender**	Female	Male	Male	Female	Male
**Age at onset (years, months)**	3 years, 6 months	17 months	3 years, 4 months	4 months	19 months
**Familial history of aHUS**	No (Sporadic)	Yes	No (Sporadic)	No (Sporadic)	No (Sporadic)
**Associated clinical manifestations at admission**					
Fever	Yes	No	Yes	No	Yes
Oligo/anuria	No	No	Yes	Yes	No
Gastrointestinal	Yes	Yes	No	No	Yes
URT infection	No	No	Yes	No	No
Hypertension	No	No	Yes	Yes	No
Biochemical parameters at admission					
Hemoglobin (g/dL)	10.7	6.7	8.2	8.8	6.1
Platelet count (x106/dL)	<10000	48000	24000	97000	117000
LDH (U/)	2725	3522	3316	781	1428
Urea (mg/dL)	112	122	117	82	89
Creatinine (mg/dL)	0.88	1.47	4.4	1.4	0.90
C3* (mg/L)	1206	1001	520	901	79.3
C4** (mg/L)	221	231	254	187	30.4
**Genetic study**					
Affected gene	CFH	CFH	C3	1) DGKε + 2) CFH	CFH
Identified mutation	Deletion in CFHR3/1	3644G > T	c.2203C > T	1) Exon 6 c.978T>G 2) CFHR3 332C>T 184G>A 1204C>T 2016A>5G 2808G>T	c.2300delA
Pattern of inheritance	Homozygosity	Heterozygosity	Heterozygosity	Homozygosity	Heterozygosity
Effect	Autoantibodies against factor H	p.Arg1215Leu	p.Arg735Trp	p.Tyr326	p.Asn767Thrfs*11
Novel mutation	No	No	No	Yes	Yes

LDH: lactic acid dehydrogenase; URT: upper respiratory tract.

* C3 normal range – 900-1800 mg/L;

** C4 normal range – 150-400 mg/L.

In 4 patients, aHUS onset followed a probable triggering event or combination of events (gastrointestinal symptoms were present in 3 cases, upper respiratory tract infection in 1, and 3 patients presented fever at admission). All patients had STEC infection excluded and negative direct coombs tests. There were no cases of seizures or altered level of consciousness. Hypertension was present in 2 patients at onset.

Two patients presented low C3 levels (minimum 79.3 mg/L, reference range 900 - 1800 mg/L), one of whom also had low C4 levels (30.4 mg/L, reference range - 150-400 mg/L). In all cases, the activity level of ADAMTS13 was confirmed to be within the normal range, a criteria required for diagnosis of aHUS.

### Genetic Analysis

Four patients (80%) were associated with CFH anomalies: one patient had heterozygous mutations in CFH, one patient presented autoantibodies against CFH associated with CFHR-related protein 3 and 1 (CFHR3/1) deletion, and two patients carried a new mutation form that has not been described so far, one of them including a single form of CFH mutation and another with CFH mutation associated with gene DGK( mutation, both homozygous; one patient carried a mutation on C3. No mutations on factor I, B, membrane cofactor protein, or thrombomodulin were found in our cohort. Patient’s genetic anomalies are described in Table 2.

**Table 2:** Acute management and long-term outcome of all included aHUS patients

	Patient 1	Patient 2	Patient 3	Patient 4	Patient 5
Acute management					
TPE	Yes	Yes	Yes	Yes	No
RRT (acute dialysis)	No	No	Yes	No	No
Outcome					
Complete remission	Yes	Yes	Yes	Yes	Yes
Chronic plasma therapy	No	No	No	Yes, during 5 years	No
Relapses (number)	No	Yes (2)	No	Yes (5)	No
Chronic hypertension	No	No	No	Yes	No
Chronic Kidney Disease	No	No	No	Yes, stage II	No
RRT (dialysis or transplant)	No	No	No	No	No
Death	No	No	No	No	No

RRT: renal replacement therapy; TPE: therapeutic plasma exchange.

### Acute Management

Four patients were treated with TPE; all received at least one session, over the course of which hemoglobin and platelet count stabilized and slowly recovered to normal. One patient needed to maintain TPE sessions until being considered in complete remission. One patient (patient 3, with a C3 gene mutation) needed RRT, with transient peritoneal dialysis (during four days). No patient was treated with eculizumab.

### Long-Term Follow-Up

After aHUS diagnosis, the median (P25-P75) follow-up period was of 136 (43.5-200.5) months. Two patients relapsed during the follow-up period; patient 2 relapsed 2 times and patient 4 relapsed 5 times; first relapses occurred 5 and 11 months after remission, respectively, and most relapses occurred after an upper respiratory tract infection. No association existed between relapse and familial history of aHUS.

One patient developed chronic hypertension and CKD stage II (eGFR 64.34 mL/min/1.73 m², at last follow-up visit). None of the patients progressed to end-stage renal disease, needing RRT. No death occurred. Data on patients’ acute management and outcome are reported in Table 2.

## Discussion

We present a Portuguese series of pediatric cases of aHUS with identified genetic anomalies, 2 of which repHUS is a relatively new entity, which emerged over the last decade as a complement dysregulation disease, with mutations in genes encoding for the main regulatory proteins of the complement pathway being identified in a growing number of patients, thanks to recent progresses in the genetic field^2^. In our cohort, the most prevalent group of mutations involved CFH which is an accordance with previous studies; in 2010, an American study reported CHF mutations in 25.3% of the cases and in 2013, a French multicenter nationwide series of cases found CHF mutations in 21.3%^10^^,^ 11 ^,^ 12.

The genetic background of aHUS continues partially unfold, and in a significant number of patients a probable causative mutation cannot be found. Several new mutations and new genes keep being reported, some in pathways not related to the complement. An example are the recently identified mutations in the gene encoding DGK(, suggesting that complement-independent forms of aHUS also exist^1^^,^ 10. In our series of cases, one patient carried a new mutation, previously not described, which included the association of a CFH mutation with a DGK( mutation, both in homozygosity, with more adverse outcome: the patient needed chronic plasma therapy for 5 years, and during the last years developed CKD. Recently published data have shown and described that the different genetic mutations known to be involved in aHUS come with a different age of disease onset, phenotype/genotype relationship, and risk of recurrence - CFH mutation is associated with higher risk of progression to ESRD after 5 years of disease. Mutations involving C3 are related with higher risk of recurrence and factor I mutations are associated with higher recurrence after kidney transplantation^10^. Knowledge of the pathological implications of complement genetic background will allow for an individualized assessment of disease predisposition and prediction of clinical evolution.

A previous study, by Noris M et al. (2010)^8^, correlating genotype and phenotype in 273 aHUS patients, reported that complete remissions were more common when membrane cofactor protein or thrombomodulin mutations were present (in 62 and 90% of cases, respectively) and that patients with membrane cofactor protein mutations also remitted spontaneously more frequently; poor responses were more frequent in patients with CFH and C3 mutations^10^. The authors also reported that the long-term outcome was somehow dependent of the genetic mutation identified, with mortality or ESRD, after initial plasma therapy, being higher among patients with CFH (77%) and complement factor I (67%) mutations^10^^,^ 11 ^,^ 12 ^,^ 13. These results reinforce the importance of performing genetic screening in all patients with suspected aHUS, to increase our understanding of the disease and the impact of each complement abnormalities on disease characteristics and progression^3^.

Recent advances facilitated the development of novel, rational treatment option targeting terminal complement activation - eculizumab, a humanized monoclonal anti-C5 antibody. Plasma therapy was the mainstay of treatment for aHUS until 2014, when an international consensus approach to the management of aHUS decided to include eculizumab, considering the safety and efficacy of this new drug^6^. In fact, at the moment, administration of eculizumab is recommended as the first line of treatment in all pediatric patients with first episode or relapsing aHUS^2^. Thus, a prompt diagnosis of aHUS at presentation is one of the most challenging task, so we can identify patients that will benefit from the treatment, allowing to start it soon as possible. The introduction of this monoclonal antibody considerably altered aHUS prognosis, allowing to reach full renal function recovery in most of the patients. When this treatment is not promptly available, TPE should be initiated within the first 24 hours, considering the poor prognosis associated with treatment delay in this disease^2^^,^ 7. TPE is also recommended when anti-factor H antibodies are identified, in combination with eculizumab treatment, with favorable outcomes both on renal function and on mortality^14^^,^ 15. In our study, 3 patients were diagnosed before eculizumab was approved for aHUS management and in the other 2 cases this monoclonal antibody was not available immediately following the diagnosis. So, in our center, we initiate TPE sessions as soon as the diagnosis hypothesis of aHUS is considered and probably this is the reason why we achieved such satisfactory results - the early initiation of TPE sessions.

The classical clinical markers (hemoglobin, platelets, LDH, and haptoglobin) and C3, C5, and functional activity of complement regulators have a limited utility to guide eculizumab dosage, as they are rough markers of complement blockade. Total complement activity (CH50) and alternative pathway complement activity (AH50) are the most commonly used tests to assess the complement activity. Treatment with eculizumab reduces CH50 activity below 10% (expected response). CH50 assay is widely available, but it has serious disadvantages as the high diversity between the different tests and a trend to the variability of CH50 in the low range, which limit its clinical utility^6^.

One of the most controversial issues concerning the use of eculizumab on aHUS is the duration of therapy. Some current recommendations suggest that eculizumab should be prescribed indefinitely. The Portuguese consensus document established in 2018 based on a worldwide consensus stated that eculizumab should be maintained for a minimum period of 6-12 months; in patients with AKI in need of RRT, eculizumab treatment is recommended for at least 3 months before establishing the final diagnosis of ESRD^2^. In 2016, Fakhouri et al. ran a multicenter multinational study, with a single-arm open-label design in adults, and concluded that in patients with no mutations or membrane cofactor protein mutations, eculizumab discontinuation can be considered; in case of patients with CFH pathogenic variants, any decision must take in consideration the high risk of relapse; and in patients with anti-factor H antibodies, eculizumab discontinuation can be considered only when titers have significantly reduced. In case of discontinuation of therapy, patients must be followed closely with regular blood and urine tests to detect relapses^16^^,^ 17 ^,^ 18.

In our study, we reported a favorable course in 4 of the 5 patients described, with only 1 patient showing progression to CKD, and no deaths. A previous study, by Fremeaux-Bacchi et al., reported higher mortality rates in children than in adults (6.7 versus 0.8%, at 1 year of follow-up) but a higher risk of progression to ESRD after the first aHUS episode in adults (46 versus 16%)^12^. Besbas et al., in 2017, published a Turkish pediatric aHUS report including 146 patients, with data collected during the 3 previous years, and reported 3 deaths and 13 patients with progression to ESKD and renal transplantation. The cohort had high frequency of MCP mutations^19^. Comparing with that report, our cohort reports a better outcome, but there are two significant differences: the size of the sample and the genetic findings. Perhaps, the racial difference between these two populations, leading to genetic background particularities, could explain this relevant fact.

Our study reports a limited number of cases and we acknowledge the importance of studying larger series of cases in order to improve our knowledge on aHUS, a complex and rare disease, which has been associated with important new findings over the course of the last years, showing us that much is yet to unveil. Identifying, describing, and understanding the genetic anomalies associated with this disease will certainly allow important improvements in terms of disease management and patients’ outcome.
